# Supplementary material for: Are carbon emissions trading and green financial instruments synergistic? -Comprehensive quantitative research based on content analysis
Source: PLoS One. 2024 Mar 7;19(3):e0298601. doi: 10.1371/journal.pone.0298601 (PMC10919601; doi:10.1371/journal.pone.0298601)
Supplement: S1 Data — (ZIP) [file pone.0298601.s001.zip › Data/地级市Z_F工作报告.zip!/║╙─╧/σo╤⌠╩╨/└┤╫╘╣1⁄2╓┌║┼í╛┬φ┐╦╩2╛▌═°í┐_2018.docx]

各位代表:

受宋殿宇市长委托，我代表市人民政府，向大会报告工作，请予审议，并请各位政协委员和列席人员提出意见。

一、过去四年工作回顾

四年来，面对复杂的经济形势和繁重的改革发展任务，在省委、省政府和市委的坚强领导下，全市上下高举习近平新时代中国特色社会主义思想伟大旗帜，深入贯彻落实党的十九大精神和习近平总书记调研指导河南时的重要讲话精神，坚持稳中求进工作总基调，以新发展理念为引领，以提高发展质量效益为中心，以推进供给侧结构性改革为主线，统筹推进“五位一体”总体布局，协调推进“四个全面”战略布局，突出“四个着力”，打好“四张牌”，扎实推进三大攻坚战，稳增长、促改革、调结构、惠民生、防风险，拼搏进取，务实重干，“十二五”规划全面完成，“十三五”规划顺利实施，富裕文明和谐美丽新濮阳建设迈出坚实步伐。

——经济实力明显增强。全市生产总值从2013年的1130.5亿元增加到2017年的1585.5亿元，年均增长9%，高于全省平均水平0.7个百分点。2017年，一般公共预算收入完成81.1亿元，是2013年的1.3倍，年均增长11%。税收占财政收入的比重保持在74%以上，位居全省前列。金融机构存贷款余额分别达1512.2亿元、744.3亿元，是2013年末的1.6倍、2倍。粮食产量52.5亿斤，连年增产增收。居民人均可支配收入18197元，年均增长10.2%，高于全省平均水平1个百分点，增幅居全省前列。市域经济综合考核保持全省第一方阵。

——发展质量不断提高。经济结构持续优化，规模以上工业增加值年均增长9.8%；第三产业增加值年均增长10.2%，2017年二三产业占生产总值的比重达到90.4%，服务业占比提高16.4个百分点。重点产业转型成效明显，石油化工产业主营业务收入突破千亿元，装备制造和食品加工产业主营业务收入分别超800亿元、700亿元，规上工业企业利润总额228.3亿元，创近年来最好水平。高新技术产业占规上工业增加值的比重31.7%，较2013年提高9.1个百分点，“六优”农产品种植面积发展到259万亩，科技对经济增长贡献率提高到54%，支撑经济增长的作用明显增强。油地一家、融合发展，战略合作进一步深化，共同推进了一批重大能源和产业项目，中石化中原天然气公司落户濮阳。

——发展优势持续积累。资源能源供给保障能力大幅提升，文96储气库建成投用，库容百亿方的文23战略储气库加快建设，中（原油田）开（封）等输气管线建成通气，丰利石化222万吨原油进口资质获批。龙丰2×60万千瓦热电机组并网发电，卫都500千伏等14座变电站建成运行。市引黄灌溉调节水库、南水北调配套、引黄入冀补淀等重大水利工程建成通水。综合交通体系建设加快推进，晋豫鲁铁路建成通车、开通客运，郑濮济高铁开工建设。南林东延、德上高速建成通车，新改建干线公路610公里、农村公路4125公里。开放平台建设实现突破，国检濮阳办事处开展业务，公用型保税仓库主体完工。城乡实现光纤、4G网络全覆盖。固定资产投资年均增长16%，民间投资年均增长13.6%，发展后劲不断增强。

——新型城镇化加快推进。第四轮城乡总体规划编制完成，土地利用总体规划获省批准。三级城镇体系进一步完善，“一中心三组团”空间发展格局基本形成，市域、县域中心城市框架持续拉大，小城镇建设有序展开。城市规划建设管理水平稳步提升，成功创建并蝉联全国文明城市、蝉联国家园林城市。2017年城镇化率43.7%，较2013年提高7个百分点，增幅全省第一。

——改革开放全面深化。“放管服”改革纵深推进，市场活力进一步释放，全市累计新登记各类市场主体11.2万户，年均增长23.4%。在全省率先完成农村承包地、宅基地和集体建设用地确权颁证，农村集体产权制度改革获“中国改革2017年度案例”。累计利用境外资金23.4亿美元、省外资金810.7亿元，年均增长9.7%、9.1%，分别高于全省平均水平4.8、0.9个百分点。进出口总值累计完成32.4亿美元，年均增长7.3%。

——环境质量持续改善。万元生产总值能耗下降27.7%、规上工业综合能源消费量下降14.6%，降幅分别高于全省平均水平2.7、4.8个百分点。2017年PM10年均浓度降至107微克/立方米，降幅居全省第1位；PM2.5年均浓度降至64微克/立方米；优良天数209天，增加28天，增幅居全省第1位，超额完成省和环保部下达的目标任务。河流出境断面水质全部达标，2017年全省水污染防治考核获得优秀。森林覆盖率达到29%，四年提高4.5个百分点。

——人民生活水平明显提高。财政民生支出累计完成701.6亿元，占一般公共预算支出的79.5%。新增城镇就业26.3万人，新增农村劳动力转移就业13万人。学前三年毛入学率、九年义务教育巩固率、高中阶段毛入学率分别达87%、97%、91.2%，超过全省平均水平。企业退休人员养老金“十三连增”。城市、农村低保标准分别提高65.4%、110%。建成各类养老服务机构87个。改造农村危房2.34万户。解决41.6万农村人口安全饮水问题，集中供水率达94.2%。80万城镇居民喝上丹江水。

四年来，我们认真贯彻以习近平同志为核心的党中央和省委省政府、市委决策部署，主要做了以下工作：

（一）力促经济平稳增长，巩固稳中向好态势

稳定经济运行。注重供给结构与需求结构有效匹配，消费和投资协同联动，着力破除要素市场配置障碍，破解实体经济发展制约和经济失衡难题，加强监测预警和调节调控，切实提高经济运行质量，出台了促进经济平稳健康发展30条、支持民营经济发展15条、稳增长保态势27个重大专项等政策措施，稳住了经济发展的基本面。

强化企业服务。深入开展“惠企政策进千企”系列活动，加强“四项对接”，发挥过桥资金和助保贷作用，累计帮助企业融资150亿元，减轻企业负担74.1亿元。惠成电子成功上市，天地人环保等10家企业新三板挂牌交易。培育高成长性企业551家，诚信钻采等7家企业进入全省百高工业企业，濮耐股份等3家企业进入全省民营企业百强。

扩大有效投资。充分发挥投资在补短板、稳增长中的关键作用，加大重大产业、基础设施项目投资力度，累计实施省市重点项目954个，完成投资4141.8亿元，435个项目建成投用，其中产业项目301个。省重点项目282个，完成投资1389.6亿元，项目数量和投资额度居全省前列。

（二）加快产业转型升级，持续提升创新力竞争力

做强做优化工产业。规划建设65平方公里新型化工基地，着力构建“一基五链三集群”发展格局，推动化工产业绿色化、循环化、智能化、高端化发展。丰利石化丙烯联产芳烃、沃森超高分子量聚乙烯等一批龙头和高端项目建成投产，工业园区与中国涂料工业协会共建全国首家绿色涂料产业园。开发区成功创建国家新型工业化产业示范基地。2017年全市规上石化企业销售收入1187亿元，是2013年的1.25倍。

改造提升传统产业。通过技术改造、战略合作、打造品牌、创新模式，推动传统产业提质增效。2017年装备制造、食品加工、现代家居、羽绒及服饰制品销售收入实现865.2亿元、755.5亿元、227.6亿元、171.5亿元，分别是2013年的1.5倍、1.7倍、2.1倍、2.2倍。濮阳成为区域性石油装备制造基地、绿色食品生产加工基地、中部家居之都、中国羽绒之乡。

培育发展新兴产业。生物基材料关键技术取得突破，形成完整产业链，聚集企业14家，我市被确定为国家生物基材料集群示范基地。天能（濮阳）循环经济产业园建成投产，成为北方最大的动力电池生产基地。建设风电场4个、集中式商业光伏电站6个，全省最大的100兆瓦光伏发电项目落户台前。新兴产业增加值年均增长16.4%。

发展壮大服务业。上项目、引龙头、提品质、扩规模，服务业发展势头强劲。华龙区特色商业区进入全省十强，跻身三星级服务业“两区”行列。2017年，全市社会物流总额突破4000亿元，增长10.2%。郑州银行、中原农险等金融机构入驻濮阳。全市旅游收入23.1亿元，是2013年的3.4倍。阿里巴巴、京东等知名电商企业入驻濮阳，百铸网、京濮电商等本土电商企业快速成长。四个县成为国家、省电子商务进农村综合示范县。

提质发展高效农业。建成高标准粮田238.5万亩、规模设施农业园区286个。培育农业产业化龙头企业197家，建成豫粮濮阳粮食产业园、木伦河冷饮产业园、汇源肉羊养殖加工、首农小优鸡养殖加工等全产业链项目。国家农业科技园区通过科技部验收。范县成功创建国家有机产品认证示范区。

（三）统筹城乡发展，推进以人为核心的新型城镇化

实施百城建设提质工程。加快构建科学合理的城镇化格局，积极探索生态、融合、适度、集约的新型城镇化之路。推进市域中心城市与濮阳县城、清丰县城、濮东工业区一体化发展。全市新建改造城市道路142条、277.5公里，敷设雨污水、供热、燃气管网3078.5公里，新建扩建污水处理厂6个。实施海绵城市项目142个、生态水系项目12个。大力推进市城区综合提升，城乡一体化示范区建设有序推进，龙山龙湖形成景观。新建改造市城区农贸市场8个。清丰县、南乐县成为全省百城建设提质工程试点。五个县城荣获国家、省级卫生县城、园林县城称号。实施棚户区改造项目110个、87111套，建成54492套，开工率、建成率居全省第一。

加快小城镇和美丽乡村建设。规划引领、完善功能、培育产业，濮城、文留等4个城镇跻身全省百强乡镇，马庄桥、张果屯、侯庙等11个镇入选全国重点镇、文明村镇和国家卫生镇。规划建设单拐红色旅游小镇、荷花小镇等特色小镇16个，岳村镇列入国家级特色小镇。城乡环卫一体化县域全覆盖。打造美丽乡村精品村81个、示范村902个，西辛庄等10个村庄入选河南最美乡村。

（四）全面深化改革开放创新，持续释放动力活力

扎实推进重点领域改革。深化行政审批制度改革，行政审批事项精简至192项。持续推进商事制度改革，实行“三十五证合一”“三十八证联办”。南乐县建成全省首家相对集中行政审批平台。深化农村综合改革，累计发放“两权”抵押贷款4.1亿元。我市被确定为全省唯一的农村集体产权制度改革整体推进试点市，华龙区在全国率先完成改革试点任务。深化投融资体制改革，组建市投资集团，总资产突破300亿元。规范PPP项目运作，开工项目21个，累计完成投资67亿元。稳步推进国企改革，市属国企由95家优化至26家，完成15家“僵尸企业”处置工作。驻濮省企“三供一业”分离移交全面完成，驻濮央企“三供一业”移交有序推进，我市被评为全省国企改革攻坚先进市。文化市场综合执法、城市管理综合执法、公交体制等改革全面完成，公立医院、养老保险、教育等改革取得阶段性成效。

持续扩大开放招商。出台开放招商25条，16个产业招商组大员上阵，挂图作战，精准招商。成功举办第十二届豫商大会、第十届海峡两岸石油化工科技经贸交流大会等重大招商活动。中建材、德力西、东方雨虹等一批知名企业落户濮阳，四年累计建成亿元以上招商项目421个。创成2个国家级、5个省级出口基地，连续三年被评为全省对外开放工作先进市。

实施创新驱动发展。培育高新技术企业、创新龙头企业、“科技小巨人”企业67家。建成省市工程技术研究中心132家、重点实验室52家、省院士工作站4家。431家企业与高校院所合作项目564项。建成国家和省质检中心6家，与清华大学等25家高校院所建立战略合作关系，建成国家和省科技企业孵化器6家，众创空间、星创天地19个。万人发明专利拥有量年均增长14.9%。我市被评为全国科技进步先进市、全国创新驱动示范市、国家知识产权试点市、全国人才工作创新最佳案例奖。

（五）着力保障改善民生，不断增强人民群众获得感

强力推进精准脱贫。把脱贫攻坚作为重大政治任务和第一民生工程，“转扶搬保救教金”七路并进，突出抓好产业扶贫、金融扶贫、搬迁扶贫等，20.28万贫困人口稳定脱贫，297个贫困村退出贫困序列，贫困发生率由10.63%降至3.58%。黄河滩区居民迁建第一批试点搬迁入住，第二批试点主体完工，三年迁建规划启动实施。2017年全国扶贫车间现场会、全省产业扶贫现场会和“千企帮千村”精准扶贫行动现场会在我市召开。2017年度脱贫工作成效考核全省第一。

全力抓好污染防治。坚持标本兼治、铁腕治污。在全省率先开展“散乱污”企业集中整治，取缔整治企业5179家。淘汰高耗能落后企业18家，拆除改造燃煤锅炉621台，削减煤炭量125万吨。完成“双替代”用户7.05万户。市城区新增供暖面积605.4万平方米，集中供热普及率83.9%，居全省第一位；5个县城区新增供暖面积423万平方米。实施“气化濮阳”工程，天然气入户率60.4%，居全省第一位。“河长制”工作全省领先。

切实做好就业社保工作。实施“阳光工程”“雨露计划”，累计培训21万人次。城镇登记失业率稳定在3%左右。我市农民工返乡创业工作走在全省前列。在全省率先实行普惠型高龄津贴政策。城乡居民医保覆盖率99%，率先实现省内异地就医即时结算。建立了困难残疾人生活补贴和重度残疾人护理补贴制度。累计发放社会救助资金13亿元，救助困难群众20多万人次。

全面发展公共事业。农村中小学“改薄”739所，新改建城区中小学142所，新增学位12.5万个。清丰县、南乐县、范县成功创建全国义务教育发展基本均衡县。新建幼儿园181所。市一高新校区建成投用。河南大学濮阳工学院实现一本招生，濮阳医专获省批复，濮阳技师学院晋升大专。乡村医疗机构标准化建设任务全面完成，全市新改建医院22所，新增床位5760张。市图书馆新馆、市工人文化宫建成投用，建成基层综合文化服务中心800个、村民服务中心1523个。全民健身活动深入开展，成功举办市九运会。

维护社会大局和谐稳定。安全生产、食品药品安全常抓不懈，连年荣获全省安全生产工作优秀市、全省食品安全工作优秀市。建设平安濮阳、法治濮阳，连年荣获全省信访工作先进市，政法机关综合执法群众满意度、公众安全感连续四年荣获全省“双第一”。2017年，我市被评为全省唯一的全国社会治安综合治理优秀市、全国宗教工作先进市、全国食品药品安全监管工作先进市。连续四年荣获全国防震减灾综合工作先进市。

四年来，我们主动接受人大及其常委会法律监督工作监督，自觉接受政协民主监督、社会和舆论监督。累计办理人大议案、代表建议1012件，吸收意见建议971条；办理政协提案1027件，吸收意见建议856条。认真听取人大代表、政协委员意见，广泛听取各民主党派、工商联、无党派人士和各人民团体意见，支持工会、共青团、妇联等人民团体依照章程开展工作。国防教育、双拥工作扎实开展，驻濮解放军和武警部队在支持地方发展、维护稳定方面做出了积极贡献。坚持依法行政，强化行政监察、审计监督，加强政府自身建设，政务环境风清气正。统计、发展研究、外事侨务、机关事务、驻外联络、公积金管理、气象、人防、史志、档案等工作都取得了新成绩。

各位代表！过去四年，是濮阳聚力发展、转型提质的四年，是辛勤耕耘、成果丰硕的四年。四年的艰辛探索、不懈奋斗，积累了弥足珍贵的经验。

这四年，我们始终坚持党对经济工作的领导不动摇，不折不扣贯彻落实中央、省委、市委决策部署，践行新发展理念，朝着更高质量、更有效率、更加公平、更可持续的方向前进；

这四年，我们始终坚持发展第一要务不偏离，把发展作为解决一切问题的关键，扩总量、提质量，心无旁骛抓落实，砥砺奋进谋宏篇；

这四年，我们始终坚持转型升级不放松，聚焦资源枯竭型城市转型主要矛盾和核心任务，精准发力、持续攻坚，重塑产业新体系；

这四年，我们始终坚持基础能力建设不懈怠，站位区域大局，前瞻谋划未来，汇资源优势、补交通短板、增发展后劲；

这四年，我们始终坚持改革开放创新不退缩，以自我革新的勇气和敢闯敢试的担当，深化改革破瓶颈、扩大开放激活力、创新驱动转动能；

这四年，我们始终坚持增进民生福祉不停步，坚持人民至上，知民情、解民忧、暖民心，一年接着一年干，锲而不舍向前走，兑现庄严承诺、落实掷地有声。

各位代表！成绩来之不易，这是省委、省政府和市委正确领导的结果，是市人大、市政协监督支持的结果，是全市人民同心同德、拼搏进取的结果。在此，我代表市政府，向广大工人、农民、知识分子，向驻濮人民解放军指战员、武警官兵、公安干警致以崇高的敬意！向关心、支持濮阳发展的社会各界人士致以衷心的感谢！

回顾过去的四年，我们也清醒地认识到，我市仍处于工业化中期、城镇化加速期，发展不充分、不平衡、不可持续的问题仍然突出，爬坡过坎、转型提质任务艰巨，工作中存在不少薄弱环节和突出问题：一是资源枯竭型城市转型面临经济下行和环保治理双重压力，行业龙头企业偏少，新兴产业增长短时难以弥补传统产业下拉影响。二是基础能力建设滞后，能源、交通瓶颈尚未根本破解，功能配套和公共服务不完善。城镇承载力、吸纳力不强，城镇化率仍低于全省、全国平均水平。三是创新能力不强，创新资源匮乏，创新投入不足，科技贡献率不高。四是三大攻坚战任务繁重，风险隐患依然存在，深度贫困区域和特殊贫困群体实现脱贫难度更大，污染防治精准持续发力不够。在住房、教育、医疗等民生领域，群众还有不满意的地方。五是政府职能需进一步转变，营商环境需进一步优化，一些工作人员能力素质和作风不适应新时代要求，一些领域不正之风和腐败问题不容忽视。我们一定要直面问题和不足，采取更加有效措施，切实加以解决，决不辜负人民群众期盼。

二、今后五年总体思路和目标任务

今后五年，是濮阳加快向高质量发展转变的关键时期，是全面建成小康社会的决胜阶段。我们正处于大有可为的历史机遇期，也面临诸多困难和挑战。

党的十九大作出了推进高质量发展的重大决策，省委发出了以党的建设高质量推动经济发展高质量、实现中原更加出彩的动员令。市委提出了推动经济高质量发展、促进濮阳由区域边缘走向中原城市群改革开放前沿、为中原更加出彩增色添彩的明确要求。以习近平同志为核心的党中央加强党对经济工作的集中统一领导，为新时代中国经济高质量发展提供了坚强的政治保障。习近平新时代中国特色社会主义经济思想，紧密结合新的时代和实践要求，明确“七个坚持”，升华了马克思主义的认识论、方法论，为推动经济高质量发展提供了根本遵循。随着国家、省重大战略深入实施，多重政策红利叠加显现；经过多年发展，我市经济实力不断壮大，经济结构持续优化，新动能加快培育，推动高质量发展具备更加坚实的物质技术基础，正值多年来最好的发展时期之一。目前，全球政治经济格局和治理体系加快重塑，国内一些长期存在的结构性矛盾和问题与外部环境冲击交织叠加，区域、行业分化态势不断加深，保持经济平稳健康发展难度加大。濮阳实现由中高速增长转向高质量发展的历史性跨越，必须统筹兼顾、谋近虑远，既要稳增长加快发展，又要转方式调优结构；既要发展城市，又要振兴乡村；既要推动经济发展，又要保护生态环境；既要发挥特色资源优势，又要补齐基础能力短板，转变发展理念，规划发展路径，明晰发展目标，以改革创新的精神和奋发有为的状态，努力开拓高质量发展新境界。

今后五年的总体思路是：以习近平新时代中国特色社会主义思想为指导，全面贯彻党的十九大精神和习近平总书记调研指导河南时的重要讲话精神，认真落实省委省政府和市委决策部署，坚持稳中求进工作总基调，坚持新发展理念，统筹推进“五位一体”总体布局、协调推进“四个全面”战略布局，按照高质量发展要求，突出资源枯竭型城市转型战略任务，加快经济发展方式转变，着力转型发展、创新发展，深化供给侧结构性改革，推动质量变革、效率变革、动力变革，构建现代产业体系；着力新型城镇化和乡村振兴双轮驱动，促进城乡均衡发展；着力深化改革开放，打造一流营商环境，激发动力活力；着力打好三大攻坚战，补齐高质量发展短板；着力推进社会事业全面发展，切实增进人民福祉，加快建设富裕文明和谐美丽新濮阳，促进濮阳由区域边缘走向中原城市群改革开放前沿，为中原更加出彩增色添彩。

根据市委七届五次全会和七届七次全会确定的奋斗目标，建议今后五年经济社会发展主要目标是：完成“十三五”规划，到2020年全面建成小康社会，在此基础上，乘势而上开启全面建设社会主义现代化新征程。

根据市委七届五次全会和七届七次全会确定的奋斗目标，建议今后五年经济社会发展主要目标是：完成“十三五”规划，到2020年全面建成小康社会，在此基础上，乘势而上开启全面建设社会主义现代化新征程。

——经济发展量质双升。在全省发展大局中的地位和作用不断提升。经济发展质量效益显著提高，资源枯竭型城市转型迈出坚实步伐，创新能力、竞争能力、可持续发展能力明显增强。在高质量发展的前提下，经济增长能快则快。财税收入稳定增长，税收占比保持全省前列。居民收入稳步提高。科技进步对经济增长的贡献率达到60%以上。

——产业转型实现突破。产业结构优化升级，服务业和新兴产业占比大幅提升，现代产业体系框架基本成型。到2020年，化工、装备制造、食品加工三大产业销售收入分别达到1500亿元、1000亿元、1000亿元以上；现代家居、羽绒及服饰制品、生物基材料三个专业化生产基地销售收入分别达到300亿元、200亿元、100亿元，在此基础上，加快向绿色化、高端化、智能化、融合化方向演进。新型化工基地综合效应突显，基础设施完备，配套能力健全，要素配置高效，资源循环利用，“一基五链三集群”发展格局基本形成。新兴产业初具规模，一批新材料、新能源、高端装备制造、生物医药企业成长壮大。现代服务业实现突破，现代物流业、信息产业、大健康产业快速发展。现代农业发展态势良好，综合效益显著提升。

——城乡发展协调并进。百城建设提质工程取得阶段性成效，市域中心城市区域战略地位得到巩固提升，开放之城、创业之城、宜居之城声名远播。县域中心城市承载、辐射作用显著增强，实力县城、活力县城、魅力县城绽放新姿。小城镇集聚效应充分发挥，一批工业重镇、商贸强镇、旅游名镇竞相发展。城镇化质量持续提升，城镇化率达到52%。现行标准下农村贫困人口全部稳定脱贫，贫困县全部摘帽。乡村振兴战略深入实施，农业供给质量明显提高，三次产业融合发展，产业支撑更加有力，生态建设、乡风文明、社会治理全面加强，乡村发展步入农业强、农村美、农民富的轨道。

——发展优势持续彰显。现代综合交通体系更加完善，郑濮济高铁建成通车，与京九高铁互联互通，融入国家高铁网络，濮阳步入高铁时代，“升”字型铁路网基本形成；“三纵三横”高速公路网建成投用。资源能源保障能力全面提升，日濮洛、鄂安沧濮阳支干线等油气管线建成投用，中原国家天然气战略储气库群建设初具规模，区域性天然气输配中心和交易中心地位日益凸显。水资源综合利用体系更加完备。开放平台日臻完善，高水平开放格局基本形成。文化资源优势充分发挥，上古文化、龙文化、杂技文化、红色文化等特色文化名片更加鲜亮。

——生态文明建设成效显著。到2020年，PM10年均浓度降至98微克/立方米以下，PM2.5年均浓度降至52微克/立方米以下，优良天数达到244天以上；25%以上全市地表水断面、50%以上国（省）考核断面水质达到或优于Ⅲ类，城市集中式饮用水水源地取水水质100%达标；全市受污染耕地安全利用率、污染地块安全率力争达到100%，在此基础上，环境质量持续改善。能源资源消耗强度大幅下降，主要污染物排放总量显著减少。省级以上湿地保护区或湿地公园实现县域全覆盖，湿地保护率达到50%以上，森林覆盖率巩固在30%以上。简约适度、绿色低碳的生活方式成为行为自觉，化为日常习惯。

——人民生活更加幸福。居民收入与生产总值同步增长，居民生活质量明显改善。社会保障全民覆盖，人人享有基本医疗卫生服务，养老服务体系加快构建，住房保障体系进一步完善，基本公共服务均等化水平稳步提高。教育现代化取得重要进展，劳动年龄人口受教育年限明显增加。就业比较充分，收入差距缩小，保持物价水平基本稳定，人民群众幸福指数明显提升。民主法治建设取得重大进展，社会治理水平不断提高，社会大局和谐稳定。

未来五年，我们充满期待、充满信心；未来五年，我们更要笃行实干、锐意进取。通过不懈努力，濮阳综合实力大幅跃升，成为中原城市群改革开放前沿、全省乃至全国重要的新型化工基地、绿色高效农业创新发展示范区、华夏历史文明传承创新区。

三、2018年政府主要工作

今年以来，按照市委经济工作会议精神，全市上下认真贯彻落实中央、省系列决策部署和王国生书记、陈润儿省长莅濮调研时工作要求，对标对表高质量发展，奋发有为、克难攻坚，经济稳中向好、质量提升，社会全面发展、和谐稳定。

经济平稳增长，上半年，全市生产总值完成807.2亿元，增长7%。三次产业比例为9.8∶56∶34.2。固定资产投资增长10.1%。社会消费品零售总额增长10.8%。质量效益提升，规上工业企业利润实现94.2亿元，增长8.4%。一般公共预算收入完成47.3亿元，增长10.8%。税收完成34.9亿元，增长12%，税收占比73.9%。转型步伐加快，工业技改投资增长7.1%，占工业投资比重16.8%。高新技术产业增加值增长16.5%，高于规上工业增速8.7个百分点，拉动工业增长4.7个百分点。第三产业投资增长12.1%。夏粮总产29.7亿斤，新增“六优”农产品种植面积14.3万亩。环境持续向好，截至6月30日，PM10平均浓度117微克/立方米，同比下降15.2%，降幅居全省第3位；PM2.5平均浓度67微克/立方米，同比下降17.3%，降幅居全省第3位；优良天数82天，同比增加14天，增幅居全省第2位。民生不断改善，民生支出完成124.6亿元，增长12%，占一般公共预算支出的78.7%。居民人均可支配收入8853元，增长9.2%。新增城镇就业2.8万人，完成省定目标的73.1%。新增农村劳动力转移就业9813人。国家卫生城市通过复审，被列为北方地区冬季清洁取暖试点市，成功创建国家森林城市。

根据高质量发展要求和今年以来经济运行态势，经济社会发展更加注重质量效益指标和约束性指标，建议今年主要预期目标为：生产总值增长7%，规模以上工业增加值增长7.5%，固定资产投资增长10%，社会消费品零售总额增长11%，一般公共预算收入增长9%，税收占一般公共预算收入比重70%，居民人均可支配收入增长9%，城镇登记失业率控制在4.5%以内，新增城镇就业3.85万人，新增农村劳动力转移就业1.5万人，常住人口城镇化率提高1.6个百分点，居民消费价格上涨控制在3%以内，PM10年均浓度低于105微克/立方米，PM2.5年均浓度低于64微克/立方米，优良天数209天以上，单位生产总值能耗降幅完成省定目标。

我们要坚持目标导向、问题导向，坚定信心、提振士气，增强责任感和紧迫感，确保完成各项任务。

（一）确保经济平稳健康发展。要把稳增长作为事关全局的突出任务来抓，落实“六稳”要求，保持经济运行在合理区间。

加强经济运行监测调控，强化要素保障，优化企业服务，落实降本减负政策，破解融资难题。支持中原石化、中原大化、丰利石化、天能集团、龙丰纸业等重点企业开足马力生产；对生产经营困难企业，因企施策，加强帮扶，促其恢复正常生产。扩大有效投资，紧紧抓住348个省市重点项目，盯节点、赶进度、早见效。研究对接国家政策，高质量谋划一批项目，争取纳入国家、省计划盘子。落实国家激发居民消费潜力的政策措施，发挥消费对经济增长的基础作用。

（二）加快产业结构优化升级。上项目、抓技改、增实力，持续推进产业提质增效。

以“三大”“三专”为重点，做强做优工业经济。化工产业，强力推进新型化工基地建设，争取总体规划、区域环评尽快获批。建成投用国家（濮阳）危险化学品应急救援中心、濮王污水处理设施及配套管网等14个项目，加快建设路网管网、集中供热等7个项目，开工建设石化产品质检中心等16个项目。切实抓好产业发展，推进展辰新型环保涂料等54个在建项目，盛通聚源3D打印材料等22个项目年内实现投产，开工建设中原石化原料结构调整项目，加快推进12个未开工项目前期工作。严格“红黄蓝绿”标识管理，改造提升可利威化工等36家企业，启动搬迁龙宇化工等78家企业，关停严重污染企业5家，为高质量项目腾出环境容量。装备制造业，重点推进东方龙机械等优势企业向智能制造、服务型制造转型。加快星光农机等项目建设，发展农机装备制造业。食品加工业，重点推进木伦河冷冻食品二期等11个项目建设，引导食品加工产业向绿色、休闲、健康、品牌方向发展。现代家居产业，重点建设广东商会家居产业园等20个项目，同步建设展销、物流、电商配套项目。羽绒及服饰制品产业，加快鹏达羽绒二期、羽绒及服饰加工产业园等项目建设。生物基材料产业，加快华乐科技可降解包装材料等4个项目建设。同时，推进风能、光能、地热能等资源综合开发利用，整合项目资源，引进关联制造业项目，重点抓好华能濮阳县50万千瓦风电场等新能源项目。

以“六大领域”为重点，大力发展服务业。现代物流业，以石化能源物流、冷链物流、快递物流、电商物流为重点，抓好中航通达物流园等6个项目建设。推进浙江传化集团公路港项目。文化旅游业，组建市文化旅游体育发展有限公司，打造区域协同旅游线路。推动杂技产业与旅游、体育深度融合。加快推进双庙体育小镇等10个重点项目。信息服务业，大数据智慧生态园主体完工，年内注册企业100家以上。积极发展金融、商贸、大健康等产业，培育总部经济、楼宇经济，推进万达广场等19个重点项目建设。

以“两大计划”为重点，不断壮大企业实力。实施龙头企业培育计划，汇聚优质资源要素，集中培育20家行业优势企业，促其成长壮大。实施企业成长促进行动计划，通过技改扩能、兼并重组、资产扩股等形式，促进个转企、小升规、规改股、股上市，催生一批高成长性企业。

（三）致力创新驱动发展。强化创新第一动力，以产业精准对接外部高端创新资源，借力借智创新发展。

扶持一批创新引领型企业。重点培育10家创新型龙头企业、3家高新技术企业、15家“科技小巨人”企业，35家科技型中小企业通过国家认定。实施30项省市科技重大专项。

培育引进一批创新引领型人才。落实人才新政20条，引进3名顶尖人才及其团队、15名高层次创新型科技人才及其团队、100名高端急需紧缺人才，招引千名英才、高校毕业生回乡就业创业。重点培养支持15名市级杰出人才、领军人才，建设3支创新型科技团队，组织开展濮阳大工匠、技术能手、功勋民营企业家等评选活动。

搭建一批创新引领型平台。新建10家市级及以上工程技术研究中心、10家企业技术中心、3家重点实验室、1家院士工作站。新建1家国家级、2家省级检验检测中心。支持濮东产业集聚区争创省级高新区。

引进培育一批创新引领型机构。加强与中科院、清华大学等合作，争取建设中科院河南科技成果转化濮阳分中心、清华大学濮阳新型化工基地联合研究机构。同时，年内发放专利权质押贷款1亿元，发放科技贷款1亿元、科技创新券5000万元以上。

（四）强化基础能力建设。牢牢抓住综合交通体系建设、资源能源保障、开放平台建设等大事要事，补齐基础能力短板，增创发展新优势。

建设现代交通体系。重点抓好“一三三”工程，完成郑濮济高铁濮阳段路基及桥梁下部工程，启动濮阳东站站房及汽车枢纽站建设；范台梁高速公路基础工程主体完工；推进濮阳至阳新、濮阳至卫辉高速公路和G240杨集、S304白堽、G342孙口黄河大桥前期工作，争取尽快开工。开工建设濮范高速与开州路互通立交。新改建“四好农村路”360公里，完成黄河滩区通道建设任务。

强化资源能源保障能力。文23战略储气库一期工程年内建成注气，推进卫11储气库前期工作。鄂安沧濮阳支干线线路贯通。日濮洛输油管线濮阳段完成30%工程量。推进濮范台输气管线、范县至东明输油管线等项目。加快卫都至塔铺500千伏线路建设。推进清丰、南乐、台前引黄调蓄工程。

加快开放平台建设。提升海关业务水平，完善公用型保税仓库功能，积极申建丰利石化原油保税仓。推进“无水港”建设，年底前完成项目选址和前期基础工作。

（五）加快新型城镇化步伐。加快农业转移人口市民化，加强精细化服务，实行人性化管理，发展产业带动就业，深化户籍制度及配套改革，推动公共服务和社会保障常住人口全覆盖，让转移人口“住得下、留得住、过得好”。

继续实施百城建设提质工程。丰富内涵发展中心城市，坚持规划引领，加强城市设计，提高城市品位。打通金堤路等3条断头路，改造提升五一路等3条道路。加快建设一污扩容工程，完成垃圾中转站、生活垃圾填埋场二期、生活垃圾焚烧发电厂年度建设任务。完成谢庄、赵娄拐等7个农贸市场新建改造任务。加快推进28个海绵城市、4条道路绿化等项目。强基提质发展县城，重点抓好路网、管网、绿系、公共服务等领域264个项目，年内完成投资210亿元。加快56个棚户区改造项目建设，年内建成17个。因地制宜发展重点镇，完善道路、污水处理等基础设施和公共服务功能，发展镇域特色经济。

提升城市管理经营水平。加快智慧城市建设，健全精细化管理长效机制，推行智能化、网格化、全天候、无缝隙管理。树立城市经营理念，盘活闲置土地，实施高效开发；综合利用公共空间、生态环境等资源。

（六）全力打好三大攻坚战。三大攻坚战是必须完成的重大政治任务，场场都是硬仗，务必打好打赢。

防范化解重大风险。防范化解金融风险，强化金融监管，堵住风险源头，打击非法集资，营造良好金融生态。防范化解企业风险，加强风险排查，分类管控，稳妥化解。探索企业市场化“债转股”。防范化解政府债务风险，规范政府举债行为，化解存量，严控增量，坚决守住不发生区域性系统性风险底线。

狠抓精准脱贫。聚焦深度贫困，突出产业扶贫、金融扶贫，确保完成5.9万农村贫困人口脱贫年度目标、实现濮阳县脱贫摘帽。扎实推进黄河滩区居民迁建，第二批试点加快搬迁，三年迁建规划首批7个乡镇安置区年内达到入住条件，2个县城安置区年内主体完工，启动搬迁工作。

强化污染防治。打好蓝天保卫战，加快产业结构、能源结构、运输结构、用地结构调整，抓好扬尘、工业企业排放、挥发性有机物专项治理。市城区新增集中供热面积200万平方米，集中供热普及率达到88%以上；县城、中心镇新增地热供暖面积180万平方米；全市新增天然气用户12万户，入户率达到71.3%。确保完成“2+26”城市秋冬季大气综合治理目标任务。打好碧水保卫战，做好黄河、金堤河等流域生态治理，开展入河排污口、非法侵占水域岸线等专项整治，加强饮用水源和地下水保护，消除城市黑臭水体。扎实推进净土保卫战，加强农业面源污染防治，开展土壤污染治理与生态修复。落实好生态环境损害赔偿制度。

（七）大力实施乡村振兴战略。按照“二十字方针”要求，落实好乡村振兴战略实施意见，编制实施五年规划，扎实做好乡村振兴各项工作。

加快产业培育，年内新增“六优”农产品种植面积30万亩以上，新建设施农业园区51个，加快标准化养驴场等7个规模化养殖项目建设。强化人才支撑，年内培训新型职业农民、农业职业经纪人1.7万人次，鼓励支持农民工、大中专毕业生、退役军人、科技人员等返乡创业。传承涵养乡村文化，加大古村落、古建筑、文化古迹保护，完成100个文明庭院示范村建设任务，培育文明乡风、良好家风、淳朴民风。改善农村生产生活环境，实施农村人居环境整治三年行动，建设20座乡镇污水处理设施、完成150所集镇公厕新建改造任务，做好4个乡镇的农村旱厕改造省级试点工作，新建美丽乡村特色村20个、示范村100个。加强农村基层组织建设，强化基层干部培训，提高综合素质和服务能力。逐步建立健全自治、法治、德治相结合的乡村治理体系。

（八）全面深化改革开放。驰而不息抓改革，坚持不懈促开放，释放动力、增添活力。

深化重点领域改革。加大“放管服”改革力度，努力实现在“一网通办”前提下“最多跑一次”。年内完成全市农村集体产权改革任务。推进国有企业改革，完成市属“僵尸企业”处置任务；年底前完成驻濮央企市政、社区、医疗机构职能移交工作。按照中央、省统一部署要求，有序推进政府机构改革等。

扩大开放招商。创新招商理念和方式，“舍得”理念引项目，利用资源引项目，专业队伍引项目，龙头带动引项目，提高招商引资实效。年内新签约、新开工亿元以上项目220个、160个以上，利用省外资金增速保持全省前列。中原油田是濮阳发展的重要战略资源，我们要持续深化战略合作，高质量谋划实施一批事关油地长远发展的大事要事，携手共创美好家园。

优化营商环境。营商环境是濮阳发展的生命线。我们要以企业需求为中心，打造一流营商环境，再创濮阳区域发展新优势。营造便利高效的政务环境，聚焦投资项目审批、商事制度、不动产登记等重点领域，减环节、优流程、砍材料、压时限，做到环节更少、时间更短、效率更高、服务更优；全面落实企业安静日制度，开展行政执法整顿活动，严厉惩治野蛮执法、任性执法、钓鱼执法，寓服务于执法之中，文明执法、规范执法、廉洁执法。营造开放公平的市场环境，放宽市场准入，鼓励支持民营企业进入金融、教育、医疗、基础设施、民生服务等领域；鼓励民营企业参与国企改革，帮助民营企业做大做强，大幅提高非公有制经济比重。营造公正透明的法治环境和诚实守信的信用环境，保护企业家合法权益，健全社会征信体系，重合同、守信用、讲诚实。构建“亲”“清”政商关系，激发和保护企业家精神，让企业家放心投资、安心经营、舒心生活。

（九）切实保障改善民生。坚持以人民为中心的发展思想，办好省市重点民生实事，让人民群众有更多的获得感、幸福感、安全感。

提高就业质量，抓好高校毕业生、就业困难人员等重点群体就业创业。完善大病保险、困难群众大病补充医疗保险和医疗救助制度。年内新改建市县城区中小学31所、农村寄宿制中小学28所。新改建普通高中6所。新改建公办幼儿园36所，扶持普惠性民办幼儿园60所。完善分级诊疗制度，推进医疗联合体、专科联盟和远程医疗建设。落实残疾儿童康复救助制度。免费开展适龄妇女“两癌”筛查、出生缺陷产前筛查、新生儿“两病”筛查和听力筛查。完成10个社区图书馆、1580个基层综合文化服务中心建设任务。加强精神文明建设，提升城市文明程度和全民文明素质。优化网络舆论生态，打造清朗网络空间。积极申办首届中国极限运动大会。

严格落实安全生产责任制，坚决遏制重特大事故发生。以创建国家食品安全示范城市为抓手，加大监管力度，保障人民群众饮食用药安全。加快社会治安防控体系建设，推进“雪亮工程”建设。切实解决信访问题。深入开展扫黑除恶专项斗争，依法打击各类违法犯罪活动，努力打造共建共治共享的社会治理格局，建设平安濮阳。

加强国防教育和国防后备力量建设，支持驻濮解放军、武警部队、预备役部队、民兵和人民防空建设，深入开展双拥共建，巩固军政军民团结。全面贯彻党的民族政策和宗教工作基本方针，促进民族团结、宗教和睦。支持工会、共青团、妇联等人民团体更好发挥桥梁纽带作用。统筹做好统计、发展研究、外事侨务、机关事务、驻外联络、公积金管理、史志、档案、防震减灾、气象、邮政、通信等工作。

四、全面加强政府自身建设

站位新时代，担当新使命，我们要牢固树立“四个意识”，坚定“四个自信”，做到“两个坚决维护”，扛起全面从严治党责任，加强政府自身建设，切实转变职能，提升履职水平，为人民提供优质高效服务。

依法行政，接受监督。严格遵守宪法法律，加快建设法治政府，把政府活动纳入法治轨道。推行政府法律顾问制度。有权不可任性，用权必受监督，依法接受市人大及其常委会的监督，自觉接受政协的民主监督，主动接受社会和舆论监督，认真听取人大代表、政协委员意见，听取民主党派、工商联、无党派人士和各人民团体意见。加强政府层级监督，做好行政复议和行政应诉工作。信守承诺，有诺必践，决不能“新官不理旧账”。做好“七五”普法工作。加强政府信息公开，主动回应社会关切，让权力运行更加公平公正、阳光透明。坚持科学决策、民主决策、依法决策，凡涉及公众利益的重大事项，都要听取社会各方意见、包括不同意见。人民政府所有工作都要体现人民意愿，效果由人民评判。

优化作风，履职担当。濮阳发展，实干为要。为人民干事是天职、不干是失职。树立干事创业良好导向，让想干事、能干事、干成事的公务人员有机会、有舞台，完善容错纠错机制，旗帜鲜明给干事者撑腰鼓劲；对庸政懒政怠政者严肃问责，让不思进取混日子的人，让表态多调门高、行动少落实差的人无位置、没市场。提高执行力，以“钉钉子”精神抓落实，定一件干一件、干一件成一件。提高广大干部政治素质和业务本领，掌握新知识、熟悉新领域、开拓新视野，善于运用法治理念、开放思维、市场方式、专业精神谋划运作、解决问题、推动工作。勇于担当，学习弘扬焦裕禄同志的“三股劲”，直面问题，敢于创新，创造出无愧于时代的业绩。

清正廉洁，干净干事。落实“一岗双责”，抓好政府系统党风廉政建设。严格执行中央八项规定及其实施细则精神，驰而不息整治“四风”，特别是力戒形式主义、官僚主义。扎牢制度笼子，拓展审计监督广度和深度，强化重点领域监管，防止违纪和腐败问题发生。巩固发展反腐败斗争压倒性态势，坚决惩治各类腐败行为。严格执行廉洁自律各项规定，廉洁修身，勤勉尽责，当好人民公仆。

各位代表！雄关漫道真如铁，而今迈步从头越。让我们更加紧密团结在以习近平同志为核心的党中央周围，在省委、省政府和市委的坚强领导下，一张蓝图绘到底，依靠全市人民，凝心聚力，苦干实干，决胜全面建成小康社会，在新时代伟大征程上，奋力谱写濮阳社会主义现代化建设新篇章！
